# Factors influencing adults’ immunization practices: a pilot survey study of a diverse, urban community in central Ohio

**DOI:** 10.1186/s12889-016-3107-9

**Published:** 2016-05-23

**Authors:** Alexa M. Sevin, Cristina Romeo, Brittany Gagne, Nicole V. Brown, Jennifer L. Rodis

**Affiliations:** Pharmacy Practice and Science, The Ohio State University College of Pharmacy, 129C Parks Hall, 500W. 12th Avenue, Columbus, OH 43210 USA; BTG Plc, BESTMSLs , Philadelphia, PA USA; Kroger Pharmacy, Columbus, OH USA; Center for Biostatistics, The Ohio State University, Columbus, OH USA

**Keywords:** Survey, Immunizations, Health behavior, Adults, Perceptions, Disparities

## Abstract

**Background:**

Adult vaccination rates in the United States are well below recommendations with disparities in race, ethnicity, and education level resulting in even lower rates for these populations. This study aimed to identify the barriers to and perceptions of immunizations in adults in an urban, underserved, multicultural community. Understanding the factors that influence adults’ decisions to receive routinely recommended vaccines will aid health care providers and public health officials to design programs to improve vaccination rates.

**Methods:**

This cross-sectional, survey-based study was conducted in January 2014 in Columbus, Ohio. Participants were recruited from four urban federally-qualified health centers and four grocery stores affiliated with those clinics. The survey gathered self-reported receipt of immunizations, knowledge about indications for immunizations, and factors influencing decisions to receive an immunization. Data was analyzed in 2014. Descriptive statistics were generated for all survey items and Chi-Square or Fisher’s Exact tests were used as appropriate to test for associations between demographic characteristics and factors influencing immunization decisions.

**Results:**

The top five factors likely to affect the decision to receive an immunization among the 304 respondents were: “doctor’s recommendation” (80.6 %), “knowing why I should get a vaccine” (78.2 %), “knowing which vaccines I need” (75.5 %), cost (54.2 %), and “concern about getting sick if I get a vaccine” (54.0 %). Significant differences in factors influencing the immunization decision exist among respondents based on ethnicity and education level. For those participants with self-identified diabetes, heart disease, or asthma, less than half were aware that certain immunizations could reduce the risk of complications associated with their disease(s).

**Conclusions:**

Data from this study may inform and shape patient education programs conducted in clinics, retailers, and communities, as well as advocacy efforts for adult immunizations. Results from this study suggest that patients would respond to programs for promoting vaccine uptake if they focused on benefits and indications for vaccines. The results also highlighted the need for education regarding immunizations for patients with chronic diseases and special indications. The differences in perceptions found between groups can be used to create targeted interventions based on the needs of those patient populations.

**Electronic supplementary material:**

The online version of this article (doi:10.1186/s12889-016-3107-9) contains supplementary material, which is available to authorized users.

## Background

Immunizations are one of the greatest public health achievements of the twentieth century. They have led to the elimination of small pox worldwide, largely eradicated polio, and are at least 94 % effective in preventing diseases such as measles, mumps, rubella, hepatitis, tetanus, pertussis, and diphtheria [1]. Despite documented effectiveness of immunizations, adult vaccination rates for routinely recommended vaccines remains suboptimal and well below Healthy People 2020 [2] recommended targets [3, 4].

While there is a need to improve adult vaccination rates ubiquitously, specific subsets of the United States population have especially low vaccination rates. Racial/ethnic disparities in immunization status among adults have been well-documented, with non-Hispanic whites having consistently higher immunization rates than other groups [3–8]. These disparities are seen in a myriad of recommended vaccinations, including influenza [3, 5–8], tetanus [4], herpes zoster [4], pneumococcal [4, 8], and human papillomavirus [4]. Disparities also exist according to education level, with those with higher education levels more likely to receive the influenza vaccine [9].

Published research shows that adults might choose not to receive a vaccination for various reasons, including belief that a healthy person does not need any vaccines [10, 11], concern about side effects [10, 11], belief that the vaccine can cause illness [8, 9, 12–14], poor attitudes toward vaccines [13], and lack of comfort or distrust in the government and health care system [13, 15]. A number of studies have also demonstrated the positive impact of a healthcare provider’s recommendation on vaccination status, with those who received a recommendation from their provider being more likely to be vaccinated [8, 10, 12, 13, 15, 16]. The influence of the cost of vaccines is less definitive, with some studies reporting an impact [13] and others reporting no impact on receipt of vaccinations [10]. One study demonstrated that higher income and insurance coverage was associated with greater vaccination rates [17]. Differences in perceptions and beliefs about vaccines can vary greatly among subsets of the population. Understanding these differences can allow for tailoring programs to specific target audiences based on their needs and concerns. Differences in attitudes toward vaccinations have been documented among various education levels [9, 11], as well as racial populations [8, 15, 18] and these differences likely contribute to disparities in immunization rates [9, 19]. Some studies have demonstrated racial disparities in vaccine uptake even when adjusting for traditional confounders such as insurance coverage, income, access to care, education, and chronic disease burden [12, 19].

While previous studies have elucidated significant differences in perceptions and barriers to vaccinations among population subgroups, they are somewhat limited in scope. Many studies included only the older adult population (≥ age 65) [8, 9, 12, 13, 18, 19] and most focused on influenza vaccination [9, 11–15, 18]. In order to improve vaccination rates for all recommended adult vaccines, health care providers and public health officials need to gain an understanding of what drives immunization practices among adults of all ages, especially among populations that experience disparities. This study aimed to expand knowledge regarding facilitators and barriers to immunizations, specifically in an urban, multilingual community. This is the first study on immunizations that takes a wider scope to identify the barriers to and perceptions of immunizations in adults of all ages in an underserved, multicultural community.

## Methods

### Participants

This cross-sectional, survey-based study was conducted in January 2014 in Columbus, Ohio. Participants were recruited in one of four urban federally-qualified health centers (FQHC), or one of four grocery stores with 340B pharmacy affiliations with those clinics. These survey sites were selected to capture participants with and without access to health care as well as those from a lower socioeconomic and culturally and ethnically diverse population. The four FQHCs are representative sites of PrimaryOne Health, a primary care safety net health center with ten locations serving the residents of Franklin and Pickaway counties in Ohio. Services provided at PrimaryOne Health include primary and specialty care, such as Obstetrics/Gynecology, Behavioral Health, Dental, Vision and more. In 2014, PrimaryOne Health served 40,000 unique patients and families generating over 110,000 encounters. The Kroger Corporation has locations in 34 states and its pharmacies filled over 175 million prescriptions during 2014. Kroger pharmacies strive to provide patients with efficient, convenient, and accurate care in accordance with the company’s mission to be a leader in the distribution and merchandising of food, health, personal care, and related consumable products and services.

### Data measures

A non-validated, 26 question survey (Additional file 2) was developed through a comprehensive literature search to deduce key factors affecting public perceptions and beliefs of immunizations. It was translated to Somali and Spanish and was reviewed by certified Spanish interpreters for language and cultural accuracy. The survey gathered demographic data, self-reported receipt of immunizations, knowledge about indications for immunizations, and factors influencing decisions to receive an immunization, including perceptions of immunization safety and effectiveness. In the survey, the word “vaccine” was used instead of “immunization,” for consistency throughout and to enhance patient understanding.

### Data collection

Participants 18 years and older were invited to complete the five-minute survey in English, Spanish, or Somali by trained surveyors. Investigators recruited patients through verbal invitation at the entrance of the grocery store or in the clinic waiting room. Surveyors were present at each grocery store for two two-hour intervals and at each clinic for two four-hour intervals, for a total of 48 surveying hours throughout the one-month study time period. Patient/customer traffic was considered in scheduling survey periods to hit high traffic time periods and also to recruit a variety of participants. Participants had the option of completing the survey on their own or having it read aloud to them by the surveyor.

### Statistical analysis

Descriptive statistics were generated for all survey items with responses expressed using frequencies and percentages. Chi-Square or Fisher’s Exact tests were used as appropriate to test for associations between demographic characteristics (specifically ethnicity and education) and factors influencing decisions to receive an immunization. Due to item non-response, all summaries and analyses are based on available data, hence the difference in sample size for various survey items. As a sensitivity analysis, we explored how associations might change by including non-response as a category for comparisons. Our findings did not indicate any changes in the conclusions reached compared to the analyses with any available data. All analyses were conducted in 2014 in SAS version 9.2 (SAS Institute, Cary, North Carolina). This study was deemed exempt by the Institutional Review Board at The Ohio State University and informed consent was obtained.

## Results

**Fig. 1:**
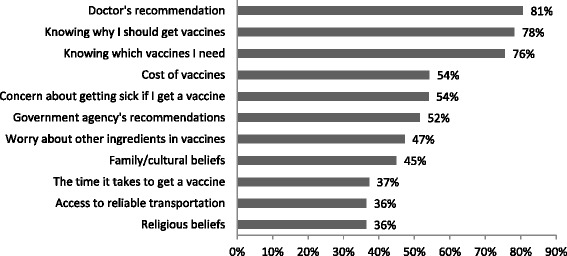
Factors somewhat or very likely to affect participants’ decision to receive a vaccine. Other factors measured include: Dislike/fear of needles (30 %), Belief that getting the disease will give me better immunity (27 %), Belief that I am healthy and do not need vaccines (27 %), Preference for alternative medicines (27 %). Note: not all categories add to total sample size owing to missing data, which ranged from 13 on “Religious beliefs” to 5 on “Worry about other ingredients in vaccines” and “Belief that getting the disease is better”

A convenience sample of 304 participants was included for the study. Demographic characteristics are reported in Table 1. The majority of the sample was female, identified as African-American, and had at least completed high school or the equivalent. Twenty-nine of 304 (9.5 %) participants filled out the survey in Spanish and no participants completed the survey in Somali. Overall, 51.5 % (155/301) reported receiving an immunization in the last year. When participants were asked about the frequency of receiving vaccines that were recommended to them, 40.9 % (123/301) reported always doing so, 39.9 % (120/301) reported sometimes doing so, and 19.3 % (58/301) reported that they never did. Figure 1 depicts the top ten factors that were somewhat likely or very likely (henceforth referred to as “likely”) to affect a participant’s decision to receive an immunization.

**Table 1:** Participants’ Demographic Information, *n* = 304^a^

Demographic Characteristic	Frequency (%)
Ethnicity	
African American	131 (43.8)
Caucasian	101 (33.8)
Hispanic	32 (10.7)
Other^b^	18 (6.0)
African	11 (3.7)
Somali	3 (1.0)
Asian	3 (1.0)
Gender	
Male	87 (30.7)
Female	194 (68.6)
Transgender: male to female	1 (0.4)
Transgender: female to male	1 (0.4)
Location	
Grocery Chain Community Pharmacy	135 (44.4)
Federally-Qualified Health Center	169 (55.6)
Annual Household Income	
Less than $10,000	85 (29.3)
$10,000 to $20,000	77 (26.6)
$20,000 to $30,000	54 (18.6)
$30,000 to $40,000	30 (10.3)
$40,000 to $50,000	20 (6.9)
Greater than $50,000	24 (8.3)
Education	
Less than High School Education	27 (9.1)
High School Education (or equivalent)	124 (41.9)
College Education	107 (36.2)
Graduate Education	38 (12.8)
Health Care	
Medicaid	86 (28.8)
Medicare	53 (17.8)
Veterans Affairs	4 (1.3)
340B	14 (4.7)
Commercial/private insurance	79 (26.4)
No insurance	82 (27.4)
Age	
18–30	82 (27.8)
31–40	59 (20.0)
41–50	50 (17.0)
51–60	64 (21.7)
61–70	32 (10.8)
> 70	8 (2.7)
Marital Status	
Single	134 (45.1)
Married	86 (29.0)
Divorced	45 (15.2)
Widowed	15 (5.1)
Separated	17 (5.7)

^a^Not all categories add to total sample size owing to missing data, which ranged from 21 on Gender to 0 on Location

^b^Other includes multi ethnic, Haitian, Indian, Native American, and West Indian

### Association between ethnicity and immunization factors

Due to small sample sizes, associations between ethnicity and factors influencing the decision to get immunized only included those participants that identified as African American, Caucasian, or Hispanic. Significant differences existed between these three ethnic groups for education level (*p* < 0.0001), with Hispanic participants more likely to have ‘Less than HS education’ compared to African American (34.4 % vs 5.5 %). African American and Caucasian groups were more likely to have college or graduate education compared to the Hispanic group (52.3 % and 53 % vs 12.5 %). Differences in insurance status also existed, with the Hispanic group more likely to report having no insurance (75.0 % vs 22.3 % vs 16.8 %, *p* < 0.001) and using the 340B program than African American or Caucasian group (15.6 % vs 3.9 % vs 3.0 %, *p* = 0.031). African American and Caucasian groups were more likely to have Medicaid (33.1 % and 27.7 % vs 3.1 %, *p* = 0.003) or commercial/private insurance than Hispanic group (26.9 % and 36.6 % vs 3.1 %, *p* = 0.001). There was no association between ethnicity and household income (*p* = 0.4129).

**Fig. 2:**
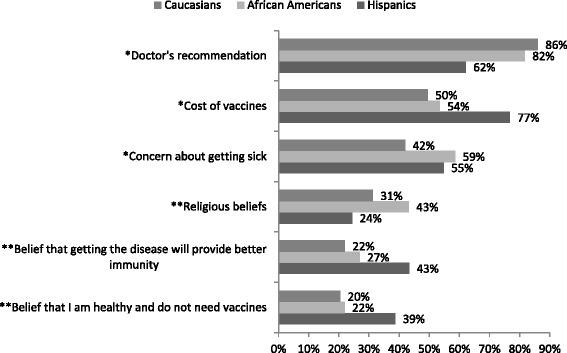
Factors somewhat or very likely to affect participants’ decision to receive a vaccine by ethnicity. ^P-values reflect association between ethnicity and likely influencing factor. *p < 0.05; ** *p* < 0.1. All other factors were not significant. Note: not all categories add to total sample size owing to missing data, which ranged from 11 on “Religious beliefs” to 4 on “Belief that getting the disease is better”

Among patients identifying as Hispanic, 31.3 % (10/32) reported that they never receive recommended vaccines, compared to 22.0 % (22/100) of Caucasians and 11.6 % (15/129) of African Americans (*p* = 0.0147). The proportion of Hispanics listing “cost of vaccines” as likely to affect their decision (76.7 %, 23/30) was significantly greater than African Americans (53.5 %, 69/129), and Caucasians (49.5 %, 49/99) (*p* = 0.030) (Fig. 2). Similar trends showed Hispanics more often indicating certain factors more likely to influence their decision to get a vaccine compared with Caucasians and African Americans such as “belief that I am healthy and do not need vaccines,” and “belief that getting the disease is better,” although these factors were not significantly associated with ethnicity. Significantly less Hispanic participants reported “doctor’s recommendation” as a factor that is likely to influence their decision to get immunized (62.1 %, 18/29) compared to African American (81.7 %, 103/126) and Caucasian participants (86.0 %, 86/100)(*p* = 0.014). Hispanic (54.8 %, 17/31) and African American populations (58.6 %, 75/128) were more likely to base their vaccination decision on “concern about getting sick if I get a vaccine” compared to Caucasians (42.0 %, 42/100, *p* = 0.042). Hispanic participants were less likely to be aware that pharmacists can give vaccines in the pharmacy without an appointment (59.4 %, 19/32) compared to African American (77.9 %, 102/131) and Caucasian participants (79.8 %, 79/99, *p* = 0.0518)

### Association between education and immunization factors

**Fig. 3:**
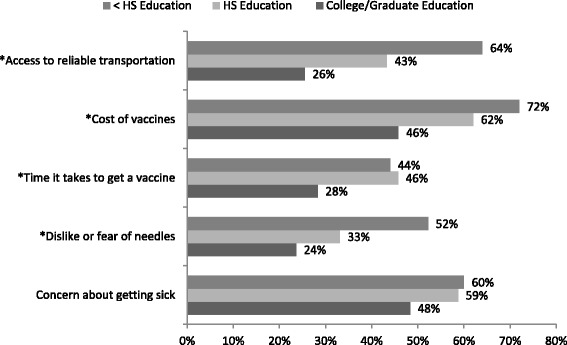
Factors somewhat or very likely to affect participants’ decision to receive a vaccine by education. ^P-values reflect association between education and likely influencing factor. *p < 0.05. Note: not all categories add to total sample size owing to missing data, which ranged from 16 on “Dislike or fear of needles” to 13 on “Concern about getting sick”

Individuals with less than a high school education were significantly more likely to indicate that access to reliable transportation, cost of vaccines, time it takes to get a vaccine, and dislike or fear of needles influenced their decision to get immunized (Fig. 3). “Concern about getting sick if I get a vaccine” was a more frequently indicated factor in the decision to be vaccinated in individuals with less than high school (60.0 %, 15/25) and high school education (58.7 %, 71/121) compared to those with college/graduate education (48.3 %, 70/145, *p* = 0.190), although this association was not statistically significant.

### Immunization education

The majority of participants (82.7 %, 249/301) indicated awareness of the existence of guidelines and recommendations for immunizations for adults. For those with diabetes or heart disease, nearly half were unaware that immunizations can reduce disease complications (46.6 %, 27/58). Of those that reported having asthma or being smokers, approximately 60 % (75/126) did not realize that immunizations can reduce their chances of contracting pneumonia. Of those with small children in the home, over one third (35.4 %, 58/164) were unaware that immunizations can decrease the risk of passing pertussis to children. The dataset supporting the conclusions of this article is included as an additional file (Additional file 1: Dataset) with this article.

## Discussion

In the urban, multicultural population captured in this cross-sectional evaluation, key facilitators and barriers to immunizations were identified. A key factor that has been identified in previous research and was confirmed in this study is that a provider’s recommendation is a strong influencing factor for receiving vaccinations [8, 10, 13], although differences in ethnicities for this factor were also found. Understanding which vaccines are needed has also been previously demonstrated as an important factor influencing individuals’ decisions to receive vaccines [10, 11]. The impact of knowledge of indication for a vaccine has not been previously studied as a factor influencing patient’s decisions, but was identified as an important factor in this study. Concern for becoming sick from a vaccine was another top factor identified in our study, which confirms previous data supporting that this apprehension may preclude patients from obtaining vaccines [8, 10, 11, 14].

While our study found “Cost of vaccine” to be in the top 5 factors influencing patients, previous studies have demonstrated conflicting results. In one study of mostly Caucasian adults age 19 and older, concern for cost of the vaccine was not a deterrent for receiving immunizations [10]. Similarly, when Santibanez et al. asked adults age 50–64 to select one of 5 reasons why they did not receive an influenza vaccination in the past year, only 3.4 % of respondents chose “vaccine costs too much.” [11] In contrast, in a systematic review focusing on adults age 65 and older world-wide, Nagata et al. found that perceived cost of the vaccine was a determinant of patient behavior on influenza vaccination [13]. These differences can be explained by examining the patient populations included and the vaccinations in question. In the study by Johnson et al., 84 % of respondents had health insurance and 70 % had an annual household income ≥ $35,000, as compared to Nagata’s study of seniors over 65 years of age who may be living on a fixed income and our study in which 69.2 % had health insurance and only 25.5 % had an annual household income ≥ $30,000 [10, 13]. In a patient population with lower household income and more uninsured patients, cost of vaccines can be a factor influencing a patient’s decision to receive a vaccination. The study by Santibanez and colleagues did not report insurance status or household income for respondents [11]. The study did focus on influenza vaccination, however, which is generally one of the lower cost vaccinations.

In this study, there was a significant difference in the number of patients reporting that they never receive recommended vaccines when examined by ethnicity. This finding has been previously reported [8]; however, these results differed in that African Americans were the least likely to report never receiving recommended vaccines. Previous studies have demonstrated that African Americans are more likely to decline vaccination than other racial/ethnic groups [8, 15, 18]. Two studies demonstrated that despite adjusting for access to care, age, education, income, and other traditional confounders for racial disparities, a racial/ethnic disparity in immunization rates persists [12, 19]. It has been hypothesized that these differences could be explained by varying perceptions and beliefs about vaccinations and the health care system in general [12, 13, 19].

This study found significant differences between ethnic groups on several factors that have not been previously examined or reported. For example, in this patient population more Hispanic patients indicated that cost of vaccine was an important factor in their decision to receive an immunization compared to African Americans and Caucasians. Although household income did not significantly differ, Hispanic patients were more likely to be uninsured, which could have impacted this factor. In addition, significantly less Hispanics in our study reported “doctor’s recommendation” as an important factor influencing their decision to receive an immunization as compared to African Americans and Caucasians. This information can certainly be useful when considering methods to improve immunization rates among certain groups. While African American and Caucasian patients in our study population may respond to a provider recommendation, this method may be less impactful on immunization rates among Hispanic patients.

Another distinction among racial/ethnic groups found in this study that aligns with previous research is the number of patients reporting concern about getting sick from the vaccine as likely to influence their decision. This factor was significantly more common among African American and Hispanic patients than Caucasian patients, similar to previous studies reported [8, 9, 11, 12]. Targeting these populations for patient education regarding this common misconception could improve immunization rates. Another potential method for reducing disparities among these groups is targeted education around access to vaccinations. Responses from this study demonstrated Hispanic patients were less likely to know that pharmacists can give immunizations in the pharmacy. Immunization marketing and education targeting the Hispanic population in our local community could enhance awareness, address concerns, and overcome access issues to immunizations for this population.

The CDC provides recommendations for vaccination for patients with heart disease, diabetes, and asthma [20] and has reported vaccination rates for influenza that lag well behind the Healthy People 2020 goals [2, 21]. Little data exists in the area of patient knowledge about need for vaccines based on these chronic disease states, as was evaluated in this survey. This study found many participants knew guidelines existed (82.7 %), but had low awareness of the benefits of vaccination in patients with asthma (40 %), as well as heart disease and diabetes (46.6 %). A study by Shoefer et al. showed 46.5 % and 14.6 % of asthma patients receiving influenza and pneumococcal vaccines, respectively [22]. Factors influencing those not being vaccinated included insufficient information and patients believing that vaccines are unnecessary [22]. These results align with responses from this study regarding insufficient patient vaccine education; patients seem to recognize vaccines are indicated, but maintain a lack of awareness of vaccine benefits. These findings may point to an opportunity to not only screen and intervene, but also to educate and empower patients so as to foster patients becoming self-advocates for vaccination and enhanced vaccination rates.

### Limitations

Limitations to this study are multifaceted. The study was limited in scope and generalizability, with a non-validated survey being conducted in Columbus, Ohio during one month with a convenience sample of participants. Due to the nature of the study, sample size calculations were not conducted; thus, investigators were unable to determine power. Participants in these settings have the potential for low health literacy. In addition, they speak different languages, which may have impacted their willingness to complete the survey and their ability to understand and adequately respond to questions. Interpreters were available during only some of the survey recruitment events. Another limitation relates to completion of surveys; some participants did not answer specific questions of the survey, which may have been due to a number of factors, including discomfort with the question or lack of understanding. Differences in socioeconomic status were not controlled for in analyses by ethnicity. While household income appeared to not be significantly different between ethnicities, this value was self-reported by patients and the survey did not collect information on the number of people per household. Responses indicated differences in education level and insurance status existed with Hispanic patients more often reporting lower education levels and a lack of insurance. These differences may or may not have contributed to their immunization practices.

## Conclusions

In a survey conducted in an urban, multicultural setting in central Ohio, factors influencing likelihood of obtaining recommended immunizations were: doctor’s recommendation, knowledge about immunizations and their effects, and immunization cost. These factors varied among ethnicities, with Hispanic participants displaying different influencing factors compared with African American and Caucasians. The data from this study may inform and shape patient education programs conducted in clinics, retailers, and communities, as well as advocacy efforts for adult immunizations in the central Ohio area. Future research opportunities exist in determining interventions that are most effective in addressing and overcoming barriers to immunizations in these populations.

## Additional file

Additional file 1: "Dataset". (XLSX 97 kb) Additional file 2: Vaccine Survey: Finding ways to help this Community Become Healthier. Additional file 2 is the survey tool used in this study. (PDF 230 kb)

## Abbreviations

FQHC: federally qualified health center
