# Supplementary material for: Factors influencing adults’ immunization practices: a pilot survey study of a diverse, urban community in central Ohio
Source: BMC Public Health. 2016 May 23;16:424. doi: 10.1186/s12889-016-3107-9 (PMC4877755; doi:10.1186/s12889-016-3107-9)
Supplement: Additional file 2: — Vaccine Survey: Finding ways to help this Community Become Healthier. Additional file 2 is the survey tool used in this study. (PDF 230 kb) [file 12889_2016_3107_MOESM2_ESM.pdf]

Vaccine Survey: Finding ways to help this Community Become Healthier

1. How often do you get vaccines that are recommended to you? Circle One:
   a) Always b) Sometimes c) Never

2. Have you received a vaccine in the last year? Yes ___ No___

3. How often do you go to the doctor's office? (circle one)

Every 1-2 months Every 3-6 months Every 7-12 months

Every 2-3 years Every 5 years Every 10 years

4. Please indicate by checking one of the boxes how much the following factors affect your decision to get a vaccine, ranging from "very likely to affect my decision" and "not at all likely to affect my decision."

	Not at all likely	Not very likely	Neutral	Somewhat likely	Very likely
A. Trust in government agency's recommendations, for example: the Centers for Disease Control (the CDC)	☐	☐	☐	☐	☐
B. My doctor's recommendation	☐	☐	☐	☐	☐
C. My religious beliefs	☐	☐	☐	☐	☐
D. My family or my cultural beliefs	☐	☐	☐	☐	☐
E. Worry about other ingredients in vaccines (chemicals or foods, such as pork or eggs)	☐	☐	☐	☐	☐
F. Knowing why I should get vaccines	☐	☐	☐	☐	☐
G. Knowing which vaccines I need	☐	☐	☐	☐	☐
H. The cost of vaccines	☐	☐	☐	☐	☐
I. The time it takes to get a vaccine	☐	☐	☐	☐	☐
J. My access to reliable transportation	☐	☐	☐	☐	☐

	Not at all likely	Not very likely	Neutral	Somewhat likely	Very likely
K. Dislike or fear of needles	☐	☐	☐	☐	☐
L. Concern about getting sick if I get a vaccine	☐	☐	☐	☐	☐
M. Belief that I am healthy and do not need vaccines	☐	☐	☐	☐	☐
N. Belief that getting the disease will give me better immunity	☐	☐	☐	☐	☐
O. Preference for using alternative / non-traditional / natural medicines	☐	☐	☐	☐	☐
P. Other: __________________	☐	☐	☐	☐	☐

Questions 5 - 17: Please check "yes" or "no" to answer the questions below.

	Yes	No
5. Are you aware that there are guidelines and recommendations for vaccines for adults?	☐	☐
6. Do you think vaccines are beneficial for you?	☐	☐
7. Do you have **diabetes** or **heart disease**?	☐	☐
8. Did you know that there are vaccines that can reduce your risk of infection and keep you out of the hospital if you have **diabetes** or **heart disease**?	☐	☐
9. Do you have **asthma** or do you **smoke**?	☐	☐
10. Did you know that vaccines can reduce the chances of **people with asthma** or **smokers** getting pneumonia, which is a bacterial infection, causes shortness of breath, requires antibiotics, and can require you to be hospitalized?	☐	☐
11. Do you have **kidney disease**?	☐	☐
12. Did you know that there are vaccines that can reduce your risk of infection and keep you out of the hospital if you have **kidney disease**?	☐	☐

	Yes	No
13. Do you have **liver disease**?	☐	☐
14. Did you know that there are vaccines that can reduce your risk of infection and keep you out of the hospital if you have **liver disease**?	☐	☐
15. Do you have small children at home, or are you around small children often?	☐	☐
16. Did you know that adults who are around small children need a vaccine to decrease the risk of passing pertussis, or whooping cough (a serious and deadly lung infection) to children?	☐	☐
17. Did you get vaccines as a child?	☐	☐
18. Did you know that pharmacists can give vaccines in the pharmacy, and no appointment is necessary?	☐	☐

19. What is the most you would be willing to pay out of pocket for a necessary vaccine? (Circle one):

$0 $10 $15 $20

20. How do you pay for health care services?

___ Medicaid (Caresource, Molina, Buckeye)
___ Medicare
___ Veterans Affairs
___ Community drug discount program or 340B
___ Commercial/private insurance
___ I have no insurance, I pay for all healthcare myself

21. What is your gender?

___ Male
___ Female
___ Transgender: male to female
___ Transgender: female to male
___ Another gender: ________

22. Education Level:

___ Less than high school education (or equivalent)
___ High school education (or equivalent)
___ College Education (or equivalent)
___ Graduate Education (or equivalent)

23.How old are you?

___ 18-30 years
___ 31-40 years
___ 41-50 years
___ 51-60 years
___ 61-70 years
___ over 70 years

24. What is your ethnicity?
Check any that apply
___ African American
___ African
___ Hispanic
___ Somali
___ Caucasian / White
___ Asian
___ Other: ________

25. Marital status:
___ Single
___ Married
___ Divorced
___ Widowed
___ Separated

26. **Yearly** Household income:
___ less than $10,000
___ $10,000 to $20,000
___ $20,000 to $30,000
___ $30,000 to $40,000
___ $40,000 to $50,000
___ greater than $50,000
